# Group Psychological Treatment Preferences of Individuals Living With Chronic Disease: Brief Report of a Saskatchewan-Based Cross-Sectional Survey

**DOI:** 10.1177/00469580241237112

**Published:** 2024-03-11

**Authors:** Kelsey M. Haczkewicz, Taylor Hill, Courtney D. Cameron, Zona Iftikhar, Natasha L. Gallant

**Affiliations:** 1 University of Regina, Regina, SK, Canada; 2 Centre on Aging and Health, Regina, SK, Canada; 3 Dalhousie University, Halifax, NS, Canada

**Keywords:** chronic disease, group therapy, mental health, patient preferences, psychological distress

## Abstract

Given that individuals with chronic diseases comorbid with psychological distress experience worse clinical outcomes than those without psychological distress, treatment of the psychological sequalae that accompanies chronic diseases is of utmost importance. Thus, the present study aimed to examine group treatment preferences among adults living with chronic disease in Saskatchewan, Canada. An online survey regarding group treatment preferences was administered to 207 participants living with chronic disease comorbid with psychological distress. The most often reported treatment scenario was virtual sessions (45%) lasting 1 h (51%) and occurring every other week (45%) in the evening (63%) for 3 to4 months (40%). Preferences included a medium group (48%), a relatively closed group nature (ie, only occasional new members; 44%), and group leadership including at least 1 professional living with chronic disease (54%). Future-oriented (81%), supportive (83%), skill-based (95%), and group discussions (78%) were desired treatment characteristics among participants. Survey results showed clear preferences on treatment content and session logistics. Slight variations exist by gender and age, but a consensus can be identified and act as a preliminary treatment plan. This study contributes to the body of literature on psychological treatment preferences for individuals living with chronic disease by outlining the preferred format and composition of groups according to those with lived experience. Group-based psychological treatment for chronic disease patients should account for these preferences to improve its acceptability and usefulness among patients.

**What do we already know about this topic?**
We know that people living with chronic disease are also at increased risk of experiencing psychological distress or having a diagnosed mental illness. We also know that Patient preferences are an important determinant of treatment outcomes, so understanding the preferences of chronic disease patients is of utmost importance.
**How does your research contribute to the field?**
This study contributes to the field by providing insights into the preferences for psychological treatment for chronic disease patients. For example, people living with chronic disease prefer virtual group sessions of about an hour in the evenings that take place over 3 to4 months.
**What are your research’s implications toward theory, practice, or policy?**
Findings can guide efforts to provide proper resources and services for people living with chronic disease and allow researchers to design and evaluate psychological treatment for chronic disease patients that best meets their needs.

## Introduction

In Canada, approximately 1 in 3 individuals are currently living with at least 1 major chronic disease.^1^ Across Saskatchewan, at least 1 in 4 residents have been diagnosed with either chronic obstructive pulmonary disease (COPD), diabetes, ischemic heart disease, or heart failure.^2^ Risk of developing chronic disease increases with age,^3^ which is particularly concerning given that, as Canadian and Saskatchewan populations continue to age, rates of chronic disease are likely to increase. Specifically, the World Health Organization^4^ has recently predicted that over two million individuals will die of a chronic disease within the next decade. It is well documented that physical health and psychological distress are highly negatively related.^5^ Psychological distress is the experience of non-specific symptoms of stress, anxiety, and depression^6^ and may lead to the development of mental illness. The incidence of mental illness is about 10% higher in those living with chronic health conditions, and 60% of physical health conditions have been documented to have a psychological component to their presentation, even without diagnosed mental illness.^7,8^

Psychological distress experienced with a chronic disease diagnosis tends to be overlooked when receiving medical care; people are generally given the choice between psychological and pharmacological treatment.^9^ Researchers have suggested that physicians are better equipped and trained to provide the biomedical aspects of care, rather than the psychological dimensions.^10^ which is concerning since those with chronic disease tend to experience psychological distress at a greater rate than the general population.^11^ Biomedical treatments have the potential to increase the risk of psychological distress in patients with chronic disease as certain treatments have been demonstrated to lead to feelings of frustration or anger, especially when compliance to treatment does not result in diminishing symptoms.^11^

Psychological distress is a common experience following the diagnosis of a chronic disease.^10^ Psychological distress may be brought on by feelings of guilt or shame that are commonly associated with the diagnosis of certain diseases, such as diabetes.^11^ The functional limitations imposed by disease may be linked to psychological distress as people need to adjust their aspirations and goals, as well as their lifestyle.^10^ However, feelings of guilt have also been linked to low physical health^12^ suggesting a bi-directional relationship rather than a causal relationship. Psychological distress may further exacerbate physical symptoms experienced as a result of chronic disease such as sleep disturbances and a lack of appetite, making it difficult to cope.^11^

Both support groups and group therapy have been demonstrated to be effective in reducing psychological distress in a variety of populations.^13,14^ Group therapy, in which individuals come together to undergo psychotherapy under the supervision of a professional, has been demonstrated to treat psychological symptoms including depression and anxiety in individuals living with Parkinson’s disease.^14^ Support groups, which differ from group therapy in that individuals come together to share their perspectives on a similar experience, have been demonstrated to be effective in reducing psychological distress and other psychological symptoms in a variety of populations. Support groups are unique in that they allow individuals to share their personal experiences in a safe and trusted environment and hear from others who are feeling and experiencing similar things. Research has demonstrated found that support groups can be used to foster a sense of control and provide information to cancer survivors, as well as improve self-care activities in COPD patients who attend in-person support groups.^13,15^ Research has also demonstrated that support groups offered virtually through social media can be effective in providing a platform for individuals living with chronic disease to obtain quick information and social support.^16^

Treatment acceptability is strongly associated with treatment preference,^17^ suggesting that accounting for preferences can increase acceptability. It is particularly important to develop feasible, acceptable, and effective ways to treat the psychological distress associated with chronic disease, as those experiencing this distress often experience difficulties in coping and negative changes in emotions, which can lead to reduced quality of life and poorer treatment outcomes.^18^ Psychological distress can act as a barrier to accessing medical treatment as it may reduce motivation to access medical care and negatively impact social relationships and social support.^18^ Additionally, psychological distress is associated with poor treatment outcomes, increases in disability and adverse physical outcomes, reduced treatment adherence, increased number of hospital visits, increased length of hospital stays, increased health service utilization, as well as increased disease burden and poorer quality of life, which only further complicate treatment.^7,8,19-21^ Taken together, evidence suggests psychological distress may reduce the quality of care received by those with chronic diseases.

Much of the literature examining the influence of patient preference on clinical outcomes in psychological treatment is limited to a choice between psychological and pharmacological treatment. In a survey of nearly 15 000 people receiving psychological treatment,^9^ the authors reported that 86% have preference for at least 1 aspect of treatment. However, information on specific preferences is sparse in the literature. For example, Myers et al (2018) found that patients valued strategies for coping with stress and negative emotions and preferred having a lay person providing treatment rather than a professional clinician.^18^ Preference for a peer to provide support has previously been reported in adults with heart disease.^19^ Given that simple logistics (eg, time, place, leader role) could influence the therapeutic value of treatment for a specific patient, identifying commonly reported preferences is valuable for design and delivery of services. Accommodating patient preferences for some aspects of treatment may lead to lower dropout rates^20,21^ and a higher perception of value,^9^ although others have reported no difference in the rate of symptom reduction for those who received their preference versus those who did not.^22^

To improve clinical outcomes of populations living with chronic disease and address the psychological distress that often accompanies chronic disease, the current study was conducted to assess what types of strategies and support individuals living with chronic diseases in Saskatchewan would like to see in a chronic disease support group. Succinctly, our research question is as follows: What are the most commonly reported psychological group treatment preferences for Saskatchewan residents living with a chronic disease?

## Method

### Participants

As the purpose of the study was to examine and support individuals living with chronic diseases in Saskatchewan, participants were deemed ineligible if (a) they did not meet the age requirements (ie, 18 years or older), (b) they resided outside of Saskatchewan, Canada, (c) they were not living with 1 or more chronic disease, or (d) they were not experiencing symptoms of psychological distress.

### Measures

#### Eligibility screener

The eligibility screener was designed to assess participant eligibility at the beginning of the survey. Participants were deemed eligible if they met the inclusion criteria of (a) at least 18 years of age (b) currently residing in Saskatchewan? (c) currently living with at least 1 chronic disease, and (d) currently experiencing psychological distress? Participants who answered “no” to any questions did not meet the inclusion criteria and were therefore deemed ineligible to participate.

#### Demographic information

Basic demographic information was collected by all eligible participants involved in the study through a survey. Information collected included age (open-ended numerical), gender (woman vs man vs other identity), racial background (open-ended text entry), and area of residence (open-ended text entry). Participants were also asked the type of chronic disease they were living with.

#### Session Logistics Preferences Questionnaire

The online survey included a 14-item self-report questionnaire adapted from the 35-item scale used in the work conducted by Kubik (2022), which investigated participants’ activity, therapist, and treatment preferences.^23^ The survey assessed preferences for participating in a chronic disease management group focused on providing coping skills and support to manage the psychological distress of individuals living with chronic disease. Participants were asked questions about their preferences for the format, frequency, length, duration, time, size, goals, and activities of the group; and how group leadership should be organized.

#### Treatment Content Preferences Questionnaire

Based on the Cooper-Norcross Inventory of Preferences,^24^ participants also indicated on a Likert scale of 1 to 7 their preferences of group structure and leadership. As disease was an open-ended answer, a basic categorical analysis was conducted to identify major diseases reported. If more than 1 disease was named, the first one was categorized.

### Procedure

Survey responses were collected from September to December of 2022. Participants were recruited using posters, social media postings, and emails asking individuals living with chronic disease and psychological distress to participate in the current study. Participants were assessed for eligibility through an online survey. Upon determining eligibility, all participants provided informed consent and completed the survey. Participants were entered in a random draw to win one of ten $25 electronic gift cards as compensation. The research study was approved by the University of Regina Research Ethics Board (#2022-094). All participants provided informed consent before completing the online survey. Data were analyzed using R (version 4.0.5). Given the exploratory nature of the study, we relied on correlational and descriptive analysis. This allowed us to generate information on preferences by gender, age, and disease.

## Results

### Demographic Information

A sample of Canadian adults (N = 207) living in Saskatchewan with chronic disease participated in this cross-sectional online survey. Our sample size of 207 participants resulted in a precision of ±0.1402. On average, participants were 46 years old, mostly women (73%), white (87%), and living in Saskatoon (33%) or Regina (23%). More detailed demographic characteristics are presented in Table 1. The most reported diseases were arthritis (33; 16%) followed by diabetes (29; 14%). The frequency of each of the 5 named diseases are reported in Table 2.

**Table 1.** Descriptive Statistics for Demographic Characteristics, N = 207.

Characteristics	N (%) or *M* (SD)
Age in years	46 (14)
Gender	
Man	47 (27%)
Woman	126 (73%)
Missing or prefer not to answer	34
Racial background	
Asian	5 (3.3%)
Black	2 (1.3%)
Indigenous	11 (7.3%)
White	131 (87%)
Missing or prefer not to answer	58
Location	
Major city: Regina, Moose Jaw, Prince Albert, or Saskatoon	118 (62.9%)
Other urban area	28 (15%)
Rural area	34 (18%)
Missing or prefer not to answer	20

**Table 2.** Frequency of Reported Diseases, N = 207.

Disease	N (%)
Arthritis	33 (19%)
Asthma	18 (10%)
Chron’s disease	9 (5.1%)
Diabetes	29 (16%)
Heart disease	13 (7.4%)
Other	74 (42%)
Missing or prefer not to answer	31

### Session Logistics Preferences Questionnaire

Frequencies for session logistics are presented in Table 3. In terms of format preference, the most often reported preference was virtual (45%) rather than in person (23%) or hybrid (33%) treatment delivery. They also wanted sessions offered every other week (45%) although weekly preference was comparable (43%). Other preferred logistics included for the session be an hour in length (51%) delivered over the longer term (9-12 weeks; 40%) in the evening (63%). Moreover, the reported preference was for group discussions (78%) of a medium size (7-9 people; 48%). There was also a preference for a relatively closed group nature (44%), with a consistent cohort of individuals involved and only new members added occasionally, although a semi-open group was preferred by almost as many (41%). Finally, participants reported a slight preference for the group leader to have the same chronic disease as them (54%) although almost nearly as many did not have a preference (46%).

**Table 3.** Frequency of Session Logistics Preferences, N = 207.

Session logistics preferences	N (%)
Format preference	
In person sessions	9 (23%)
Mix of in person and virtual	13 (33%)
Virtual sessions	18 (45%)
Missing or prefer not to answer	167
Frequency preference	
2 times per month	79 (45%)
4 times per month	74 (43%)
8 times per month	21 (12%)
Missing or prefer not to answer	128
Length preference	
Less than an hour	44 (22%)
1 h	101 (51%)
1.5 h	33 (17%)
2 h	19 (9.6%)
2.5 h	0 (0%)
Over 2.5 h	1 (0.5%)
Missing or prefer not to answer	9
Duration preference	
Short term (1-4 weeks)	45 (24%)
Medium term (5-8 weeks)	66 (36%)
Long term (9-12 weeks)	73 (40%)
Missing or prefer not to answer	23
Time of day preference	
Morning	18 (11%)
Afternoon	44 (27%)
Evening	104 (63%)
Missing or prefer not to answer	41
Goals preference	
Completing assigned readings	13 (6.4%)
Having group discussions	158 (78%)
Listening to presentations	6 (3.0%)
Participating in role plays	9 (4.4%)
Writing personal reflections	17 (8.4%)
Missing or prefer not to answer	4
Size preference	
Small, 4-6	60 (32%)
Medium, 7-9	91 (48%)
Large, 10-12	38 (20%)
Missing or prefer not to answer	18
Group members preference	
Completely open	28 (14%)
Relatively closed	87 (44%)
Semi-open group	81 (41%)
Missing or prefer not to answer	11
Diagnosis of group members preference	
Others need not have same disease	89 (46%)
Others should have same disease	105 (54%)
Missing or prefer not to answer	13

### Treatment Content Preferences

For treatment content preferences, the means and standard deviations for each treatment content preference is presented in Table 4. Furthermore, the most commonly reported treatment content preferences included group leaders that taught skills to deal with problems (95%), were supportive (83%), focused on the future (81%), encouraged individuals to express strong feelings (79%), and encouraged individuals to get into difficult emotions (73%).

**Table 4.** Means and Standard Deviations of Treatment Content Preferences, N = 207.

Treatment content preferences	*M* (SD)
Focus on specific goals	5.21 (1.53)
Give structure to the group	5.02 (1.78)
Teach me skills to deal with my problems	5.97 (1.14)
Give me homework to do	3.76 (2.16)
Take a lead in the group	3.96 (2.10)
Encourage me to get into difficult emotions	4.81 (1.84)
Encourage me to express strong feelings	5.02 (1.71)
Focus mainly on my feelings	4.36 (1.78)
Be gentle	4.00 (2.16)
Focus in my life in the past	2.90 (1.97)
Help me reflect on my childhood	3.18 (1.96)
Focus on my future	5.47 (1.53)
Be supportive	5.32 (1.77)
Not interrupt me	3.64 (2.10)
Not be challenging on my beliefs and values	3.51 (2.21)
Support my behavior unconditionally	3.01 (2.11)

*Note.* All questions are asked on a Likert-type scale ranging from 1 to 7.

**Figure 1.**
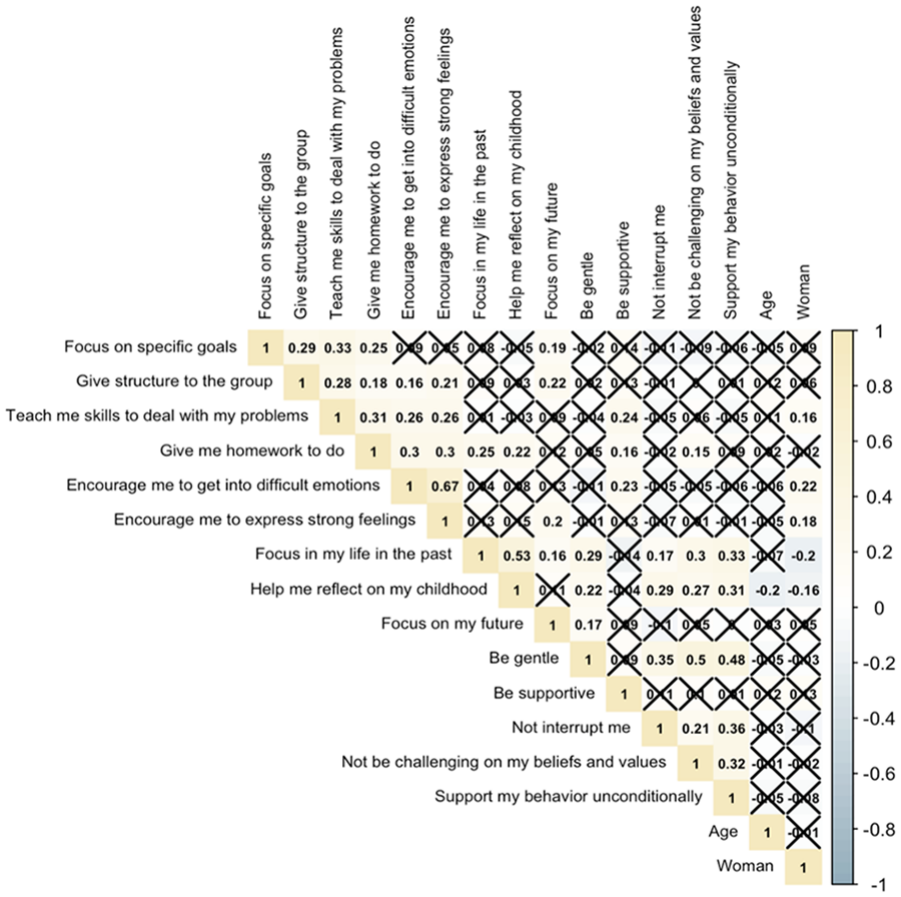
Bivariate correlations between age, gender, and treatment content preferences.

Bivariate correlations between age, gender, and treatment content preferences are presented in Figure 1. Age was significantly negatively associated with preferring not to reflect on childhood in treatment (*r* = −.20, *P* < .05). Women preferred treatment that taught skills to deal with problems (*r* = .16, *P* < .05) and provided encouragement to get into difficult emotions and express strong feelings (*r* = .18, *P* < .05). On the other hand, men preferred not to focus on the past (*r* = −.20, *P* < .05) or their childhood (*r* = −.16, *P* < .05).

Significant correlations were also observed between treatment content preferences, and those correlations related to the most commonly reported treatment content preferences are described. Participants who preferred treatment that taught skills to deal with problems similarly preferred a supportive group leader (*r* = .24, *P* < .05) and treatment that focused on specific goals (*r* = .33, *P* < .05), gave structure to the group (*r* = .28, *P* < .05), gave them homework to do (*r* = .31, *P* < .05), encouraged them to get into difficult emotions (*r* = .26, *P* < .05), and encouraged them to express strong feelings (*r* = .26, *P* < .05). Participants who preferred a supportive group leader also tended to prefer treatment that gave them homework to do (*r* = .16, *P* < .05) and encouraged them to get into difficult emotions (*r* = .23, *P* < .05). Participants who preferred treatment that focused on the future tended to also prefer treatment that encouraged them to get into difficult emotions (*r* = .20, *P* < .05) in addition to focusing on specific goals (*r* = .19, *P* < .05) and giving structure to the group (*r* = .22, *P* < .05). Participants who preferred treatment that encouraged them to get into difficult emotions and express strong feelings tended to prefer treatment that gave structure to the group (*r* = .16, *P* < .05; *r* = .21, *P* < .05) and gave homework to do (*r* = .30, *P* < .05).

## Discussion

The overall objective of the current study was to determine the session logistics and treatment content that individuals living with chronic disease(s) would like to be offered in a chronic disease group to improve their symptoms of psychological distress. Survey results showed clear preferences for both session logistics and treatment content.

Women living with chronic disease indicated their preference for being encouraged to get into difficult emotions and express strong feelings from the group leader. In general, women tend to prefer psychotherapy more than men,^25^ and they tend to express and talk about their feelings more easily than men,^26^ who noted their preference for not reflecting on the past or their childhood. Khalsa et al report that men prefer psychological treatment less than their women counterparts, and that patients preferring psychotherapy tend to report having childhood issues to work through.^27^

Individuals living with chronic disease who preferred structure to the group also wanted to learn skills, be assigned homework to practice those skills, be encouraged to explore their feelings, and focus on the future. Learning strategies to manage stress can be considered an important life skill and is a common preference in the psychological treatment literature.^19^ A review of community mental health promotion program facilitators has shown that having the opportunity to practice these skills through homework is linked to improvements in mental health.^28^ In general, those who valued focusing on the future also indicated preference for the group leader to be gentle. These preferences may be connected through humanistic psychotherapy, which includes future-oriented therapy^29^ and unconditional positive regard from the therapist to client.^30^

Older participants, relative to younger participants, preferred not to reflect on childhood. Being prepared for future care (a form of health literacy)^31^; is linked to better mental health outcomes.^32^ This future-oriented approach to health is considered proactive coping^33^ and is particularly relevant for older adults.

With regards to session logistics, participants preferred virtual weekly evening sessions that lasted an hour in length over a few months. Younger people tend to report a preference for virtual care,^25^ particularly over the internet.^19^ Moreover, time of day is a particularly important treatment characteristic; in a survey of nearly 15 000 people receiving psychological treatment, nearly three-quarters of patients expressed preference for time of day of treatment.^9^

In the present study, participants wished to participate in group discussions with a medium-sized group that is relatively closed and with members who have the same disease. Participating in group therapy with peers who share the same diagnosis is commonly reported^18,19^ and considered peer support. Interestingly, participants also preferred to learn skills for managing distress that is future-focused, which may be specific to trained clinicians who can offer that opportunity. On the other hand, desiring to explore emotions and difficult feelings seems to fit with the preference for peer support and group discussion with peers who have similar experiences.

While the results of the present study are promising in informing the creation of a group psychological treatment for those with chronic disease, empirically-supported treatments need to be considered in addition to participant preferences. Empirically-supported guidelines for providing psychological treatment should always be considered as it can be used to determine which treatment, circumstance, and provider will likely yield the greatest results.^34^ Evidence-based interventions which have been tested on multiple populations can be effective in reducing mental health symptoms in individuals living with chronic disease. However, the preferences of individuals living with chronic disease may be crucial in the creation of a group treatment for several reasons, Individuals living with chronic disease may be limited in their ability to access treatment due to mobility or transportation barriers, withstand the length of the treatment session due to pain, or participate in certain treatment components. For these reasons, it is crucial that the preferences of end-users are considered and implemented as best as possible within empirically-supported treatments. Additionally, some individuals living with chronic disease may benefit from group support, rather than group therapy. While some individuals may require clinical intervention through group therapy provided by a mental health professional, others may yield the greatest results through receiving group support from peers.

## Strengths and Limitations

Although the current study found several valuable findings, the results should be interpreted while accounting for a few limitations. First, the sample size was modest and consisted of primarily white women. This lack of racial and gender diversity within the sample may be a potential limitation to the generalizability of the study. Second, the R-values for our correlations were weak to moderate. Third, most of the survey respondents were diagnosed with arthritis as their chronic disease, thus limiting the generalizability of the study. Despite these limitations, the study has several notable strengths. To our knowledge, this study was the first of its kind to assess chronic disease group preferences for a Saskatchewan-based population. The findings of this study will also guide future research aimed at assessing proper resources and services for individuals living with chronic disease. From a practical standpoint, the findings of this study will allow researchers to develop a support group best suited for those vulnerable to the psychological distress associated with their chronic disease.

## Conclusion

This study evaluated the types of strategies and support individuals living with chronic diseases in Saskatchewan would like to see in a chronic disease support group. Overall, our results convey that those living with chronic disease believe they would benefit from a support group designed to improve their experiences of chronic disease. Participants indicated the ideal session logistics would include virtual weekly evening sessions that lasted an hour over 3 to4 months. The group leader role should be supportive, future-oriented, encourage emotion- and feeling-based work, and teach skills to deal with problems. Women value encouragement to get into difficult emotions and express strong feelings. Older participants and men preferred to not reflect on childhood. Those who preferred structure to the group also valued skill-learning, getting homework, being encouraged about feelings, focusing on future. These findings will be used to inform the creation of a chronic disease group, which will be further empirically investigated, and if effective, scaled up for individuals living with chronic disease across Canada.

## Supplemental Material

sj-docx-1-inq-10.1177_00469580241237112 – Supplemental material for Group Psychological Treatment Preferences of Individuals Living With Chronic Disease: Brief Report of a Saskatchewan-Based Cross-Sectional Survey Supplemental material, sj-docx-1-inq-10.1177_00469580241237112 for Group Psychological Treatment Preferences of Individuals Living With Chronic Disease: Brief Report of a Saskatchewan-Based Cross-Sectional Survey by Kelsey M. Haczkewicz, Taylor Hill, Courtney D. Cameron, Zona Iftikhar and Natasha L. Gallant in INQUIRY: The Journal of Health Care Organization, Provision, and Financing
