# Supplementary material for: Group Psychological Treatment Preferences of Individuals Living With Chronic Disease: Brief Report of a Saskatchewan-Based Cross-Sectional Survey
Source: Inquiry. 2024 Mar 11;61:00469580241237112. doi: 10.1177/00469580241237112 (PMC10929047; doi:10.1177/00469580241237112)
Supplement: sj-docx-1-inq-10.1177_00469580241237112 – Supplemental material for Group Psychological Treatment Preferences of Individuals Living With Chronic Disease: Brief Report of a Saskatchewan-Based Cross-Sectional Survey [file sj-docx-1-inq-10.1177_00469580241237112.docx]

**Eligibility Screener**

Thank you for your interest in this study! Before you can proceed, we need to make sure that you are eligible for this study. Please answer the following question(s).

1. Are you at least 18 years of age?
   - Yes 🡪 Proceed to B
   - No 🡪 Prospective participants thanked for their time and study discontinued
2. Do you live in Saskatchewan?
   - Yes 🡪 Proceed to C
   - No 🡪 Prospective participants thanked for their time and study discontinued
3. Are you currently living with at least one chronic disease? A chronic disease is any disease that lasts at least one year. Some of these diseases include arthritis, asthma, cancer, chronic obstructive pulmonary disease (COPD), diabetes, epilepsy, heart failure, ischemic heart disease, and stroke. These are not the only chronic diseases, so if one or more of your chronic diseases are not listed above, we still encourage you to respond ‘Yes’ to this question.
   - Yes 🡪 Proceed to Participant Consent Form
   - No 🡪 Prospective participants thanked for their time and study discontinued

**Participant Consent Form**

**Project Title:** Mental Health Group for People Living with Chronic Disease in Saskatchewan

**Researchers:**  Courtney Cameron

Research Coordinator, Aging, Residents, and Caregivers (ARC) Research Unit

Kelsey Haczkewicz

Research Assistant, Aging, Residents, and Caregivers (ARC) Research Unit

Student, Department of Psychology

Donna Dumitrescu

Research Assistant, Aging, Residents, and Caregivers (ARC) Research Unit

Student, Department of Psychology

**Supervisor:**  Natasha Gallant, Ph.D.

Director, Aging, Residents, and Caregivers (ARC) Research Unit

Assistant Professor, Department of Psychology

**Mailing Address:** Department of Psychology

University of Regina

3737 Wascana Parkway

Regina, SK

S4S 0A2

**Phone Number:** (306) 337-3227

**Email Address:** [arcresearch@uregina.ca](mailto:arcresearch@uregina.ca)

**Website:** [www.arcresearch.ca](http://www.arcresearch.ca)

***What is the purpose of this study?***

The aim of this study is to better understand the preferences of people living with chronic disease(s) in Saskatchewan. A chronic disease is any disease that lasts at least one year. Some of these diseases include arthritis, asthma, cancer, chronic obstructive pulmonary disease (COPD), diabetes, epilepsy, heart failure, ischemic heart disease, and stroke, but these are not the only chronic diseases. More specifically, we are looking to understand preferences for a group focused on providing coping strategies and support that helps people manage their mental health while living with chronic disease(s). This study has been funded by an Establishment Grant from the Saskatchewan Health Research Foundation.

***What does participation in this study involve?***

In this survey for a chronic disease group, we will ask you about your chronic disease(s), a few pieces of information about you (e.g., age, gender), and your preferences for the new group that we are designing. If you do not wish to answer one or more question(s), you can click ‘Prefer not to answer’ to skip the question(s). It should take you no longer than 1 session of up to 10 minutes to complete the survey. Please note that the survey must be completed in one sitting or be reopened in the same browser to make sure no data is lost. Once you have ended the quick survey for a chronic disease group, you will be taken to an exit survey. Here, you can provide us with your first name and email address to be entered into a draw for a chance to win 1 of 10 gift cards of $25. This exit survey will not be attached to your answers in the quick survey on a chronic disease group in any way. Once the random draw is complete and winners of the gift cards have received their gift card of $25, all contact information will be permanently deleted.

***What are the potential risks of participating in this study?***

Given the nature of the topic under study, some of the questions asked may be distressing to participants. If you experience significant distress during of following your participation in this study, we encourage you to call your local crisis helpline or emergency department. Additional resources include Wellness Together Canada (<https://wellnesstogether.ca/en-CA>), the Canadian Mental Health Association (<https://cmha.ca/>), and the Mental Health Commission of Canada (<https://www.mentalhealthcommission.ca/>). These resources will be provided at the end of the study.

***What are the potential benefits of participating in this study?***

Findings from this study will be used to inform the design of a mental health group for people living with chronic disease(s) in Saskatchewan. However, please note that no personal benefits from participation are guaranteed. As a token of our appreciation, you will also be entered in a draw to win 1 of 10 gift cards of $25.

***Will my participation in this study be kept anonymous and confidential?***

The study’s researchers and supervisor will be the only persons with access to the data and these persons will employ the strictest rules of anonymity and confidentiality. Electronic data will be kept in password-protected databases on password-protected computers. To keep your electronic data confidential, it is strongly recommended that you complete the survey on a private (rather than public) computer and/or server as well as clear your browser history and/or cache following survey completion. To ensure anonymity, your identifying information (i.e., first name, email address) will be kept separate from your responses to the survey. That is, at the end of the study, you will be redirected to an exit survey to enter your first name and email address to be entered in the draw. The use of email as a form of communication is not secure because emails are not encrypted and are therefore vulnerable to interception from outside sources. We will keep email communication to a minimum and any emails related to your participation in this study will have a non-descriptive subject line (i.e., CD Group). Furthermore, to comply with the University of Regina’s policies and procedures, participants who receive one of the gift cards from the draw will be required to sign a Participant Receipt of Compensation Form. Participant Receipt of Compensation Forms will be stored in a sealed envelope in a secure file within the Financial Services department at the University of Regina. The envelope will only be opened if the file is part of a financial audit. The only information in the file will be your name and the type and amount of compensation you received. Data from this study will be published in peer-reviewed journal articles and on the ARC Research Unit website as a summary as well as presented at academic conferences and community events, but only group results will be presented. All data will be stored for no less than seven years and no more than ten years following publication in a peer-reviewed journal article. When it is time to destroy the data, electronic data will be permanently deleted.

***What happens if I decide to withdraw my content to participate in the study?***

We hope that you will assist us with this study; however, your decision to participate is completely voluntary. You can choose to answer only those questions that you are comfortable answering by selecting the “Prefer not to answer” option. You may withdraw from the study at any time and for any reason without a need for explanation and without penalty of any sort. If you wish to withdraw from completing the survey, please click the “End Survey” button at the bottom of the survey. You will then be redirected to the exit survey where you can provide your first name and email address to be entered into a draw. Once you enter your first name and email address, please close your web browser to exit the survey. Due to the anonymous nature of the survey, your data cannot be withdrawn from the study after it has been collected. Therefore, partial responses to the survey will be kept.

***What will happen after this study?***

Results of this study will be available by the end of April 2023. If you would like to receive a summary of these results, you may request a copy of the results from the Researchers or Supervisor. Please note that reaching out to the Researchers or Supervisor will result in your identity no longer being anonymous. If you wish to remain anonymous, you are welcome to access a summary of the results the Supervisor’s website at [www.arcresearch.ca](http://www.arcresearch.ca).

***Who do I contact if I have any questions or concerns?***

This project was reviewed and received approval through the Research Ethics Board at the University of Regina on […]. If you have any questions, or would like more information about the study, please contact the Researchers or Supervisor at [arcresearch@uregina.ca](mailto:arcresearch@uregina.ca) or (306) 585-4219. If you have any questions or concerns about your rights as a research participant, you may contact the Chair of the University of Regina’s Research Ethics Board at (306) 585-4775 or at [research.ethics@uregina.ca](mailto:research.ethics@uregina.ca). Participants may call the Research Office’s toll-free number at (866) 966-2975.

***How do I provide my consent to participate in this study?***

If you have read and understood the information in the Participant Consent Form, and are still interested in participating in this study, then you can proceed to the survey. By completing and submitting the survey, your free and informed consent is implied and indicated that you understand the above conditions of participation in this study.

**Chronic Disease Group Survey**

We are looking to design a new group for people living with chronic disease(s) in Saskatchewan. This group will focus on providing coping skills and support that helps you manage your mental health while living with chronic disease(s). Before we start designing the group, we want to hear from people like you. That’s why we designed this quick survey.

In this quick survey for a chronic disease group, we will ask you about your chronic disease(s), your preferences for the new group that we are designing, and a few pieces of information about you (e.g., age, gender). If you do not wish to answer one or more question(s), you can click ‘Prefer not to answer’ to skip the question(s).

Finally, once you have ended the quick survey for a chronic disease group, you will be taken to an exit survey. Here, you can provide us with your first name and email address to be entered into a draw for a chance to win 1 of 10 gift cards of $25. This exit survey will not be attached to your answers in the quick survey on a chronic disease group in any way.

1. **What chronic disease(s) are you currently living with?** A chronic disease is any disease that lasts at least one year. Some of these diseases include arthritis, asthma, cancer, chronic obstructive pulmonary disease (COPD), diabetes, epilepsy, heart failure, ischemic heart disease, and stroke. These are not the only chronic diseases, so if one or more of your chronic diseases are not listed above, we still encourage you to add it to the list below.
   - ____________________
   - ____________________
   - ____________________
   - ____________________
   - ____________________
   - Prefer not to answer

As previously mentioned, we are designing a new group in Saskatchewan to help people living with chronic disease(s) better manage their mental health. We are interested in hearing about your preferences for this group.

1. **What is your preference for the format of the group?**
   - In-person sessions
   - Virtual sessions
   - Mix of in person and virtual sessions
   - Other: ____________________
   - Prefer not to answer
2. **What is your preference for the frequency of the group?**
   - 1 time per week
   - 2 times per week
   - 1 time per 2 weeks
   - Other: ____________________
   - Prefer not to answer
3. **What is your preference for the length of the group sessions?**
   - Less than 1 hour
   - 1 hour
   - 1.5 hours
   - 2 hours
   - 2.5 hours
   - More than 2.5 hours
   - Other: ____________________
   - Prefer not to answer
4. **What is your preference for the duration of the group?**
   - A short-term group of about 1 to 4 weeks
   - A medium-term group of about 5 to 8 weeks
   - A long-term group of about 9 to 12 weeks
   - Other: ____________________
   - Prefer not to answer
5. **What time of day would you prefer to attend this group? Select all that apply.**
   - Weekday mornings
   - Weekday afternoons
   - Weekday evenings
   - Weekend mornings
   - Weekend afternoons
   - Weekend evenings
   - Other: ____________________
   - Prefer not to answer
6. **What goal(s) would you prefer to be the focus of the group? Select all that apply.**
   - Learning coping skills for dealing with chronic disease
   - Discussing existential topics related to chronic disease such as life’s purpose or spirituality
   - Emphasizing hope and change while living with chronic disease
   - Receiving emotional support for chronic disease
   - Obtaining medical education about chronic disease and its treatment
   - Other: ____________________
   - Prefer not to answer
7. **What activities would you prefer to be the focus of the group? Select all that apply.**
   - Having group discussions
   - Listening to presentations
   - Writing personal reflections
   - Completing assigned readings
   - Participating in role plays
   - Watching videos
   - Other: ____________________
   - Prefer not to answer
8. **What is your preference for the size of the group?**
   - Small group of 4 to 6 persons
   - Medium group of 7 to 9 persons
   - Large group of 10 to 12 persons
   - Other: ____________________
   - Prefer not to answer
9. **What is your preference for who is included in the group each time?**
   - An open group that includes some of the same members and new members each time
   - A relatively closed group that includes most of the same members each time with new members on occasion
   - A completely closed group that includes the same members each time
   - Other: ____________________
   - Prefer not to answer
10. **What is your preference for who is included in the group based on chronic disease diagnosis?**
    - Other group members need to have the same chronic disease(s) as me
    - Other group members do not need to have the same chronic disease(s) as me
    - Other: ____________________
    - Prefer not to answer
11. **What is your preference with regards to group leadership? Select all that apply.**
    - A group led by one or more professionals who are not living with chronic disease(s)
    - A group led by at least one professional living with chronic disease(s)
    - A group led by at least one peer living with chronic disease(s)
    - Other: ____________________
    - Prefer not to answer
12. **On each of the items below, please indicate your preferences for how the group leader should work with you by selecting the most appropriate response.** **A 3 indicates a strong preference in that direction, 2 indicates a moderate preference in that direction, 1 indicates a slight preference in that direction, 0 indicates no preference in either direction or an equally strong preference in both directions.**
    - I would like the group leader(s) to...

| Focus on specific goals | | |  | Not focus on specific goals | | |
| --- | --- | --- | --- | --- | --- | --- |
| 3 | 2 | 1 | 0 | -1 | -2 | -3 |

** Prefer not to answer*

| Give structure to the group | | |  | Allow the group to be unstructured | | |
| --- | --- | --- | --- | --- | --- | --- |
| 3 | 2 | 1 | 0 | -1 | -2 | -3 |

** Prefer not to answer*

| Teach me skills to deal with my problems | | | Not teach me skills to deal with my problems | | | |
| --- | --- | --- | --- | --- | --- | --- |
| 3 | 2 | 1 | 0 | -1 | -2 | -3 |

** Prefer not to answer*

| Give me ‘homework’ to do | | |  | Not give me ‘homework’ to do | | |
| --- | --- | --- | --- | --- | --- | --- |
| 3 | 2 | 1 | 0 | -1 | -2 | -3 |

** Prefer not to answer*

| Take a lead in the group | | |  | Allow me to take a lead in the group | | |
| --- | --- | --- | --- | --- | --- | --- |
| 3 | 2 | 1 | 0 | -1 | -2 | -3 |

** Prefer not to answer*

| Encourage me to go into difficult emotions | | | | Not encourage me to go into difficult emotions | | | |
| --- | --- | --- | --- | --- | --- | --- | --- |
| 3 | 2 | 1 | 0 | | -1 | -2 | -3 |

** Prefer not to answer*

| Encourage me to express strong feelings | | | | Not encourage me to express strong feelings | | | |
| --- | --- | --- | --- | --- | --- | --- | --- |
| 3 | 2 | 1 | 0 | | -1 | -2 | -3 |

** Prefer not to answer*

| Focus mainly on my feelings | | |  | Focus mainly on my thoughts | | |
| --- | --- | --- | --- | --- | --- | --- |
| 3 | 2 | 1 | 0 | -1 | -2 | -3 |

** Prefer not to answer*

| Focus on my life in the past | | |  | Focus on my life in the present | | |
| --- | --- | --- | --- | --- | --- | --- |
| 3 | 2 | 1 | 0 | -1 | -2 | -3 |

** Prefer not to answer*

| Help me reflect on my childhood | | |  | Help me reflect on my adulthood | | |
| --- | --- | --- | --- | --- | --- | --- |
| 3 | 2 | 1 | 0 | -1 | -2 | -3 |

** Prefer not to answer*

| Focus on my past | | |  | Focus on my future | | |
| --- | --- | --- | --- | --- | --- | --- |
| 3 | 2 | 1 | 0 | -1 | -2 | -3 |

** Prefer not to answer*

| Be gentle | | |  | Be challenging | | |
| --- | --- | --- | --- | --- | --- | --- |
| 3 | 2 | 1 | 0 | -1 | -2 | -3 |

** Prefer not to answer*

| Be supportive | | |  | Be confrontational | | |
| --- | --- | --- | --- | --- | --- | --- |
| 3 | 2 | 1 | 0 | -1 | -2 | -3 |

** Prefer not to answer*

| Not interrupt me | | |  | Interrupt me and keep me focused | | |
| --- | --- | --- | --- | --- | --- | --- |
| 3 | 2 | 1 | 0 | -1 | -2 | -3 |

** Prefer not to answer*

| Not be challenging of my beliefs and views | | |  | Be challenging of my beliefs and views | | |
| --- | --- | --- | --- | --- | --- | --- |
| 3 | 2 | 1 | 0 | -1 | -2 | -3 |

** Prefer not to answer*

| Support my behaviour unconditionally | | | Challenge my behaviour if they think it’s wrong | | | |
| --- | --- | --- | --- | --- | --- | --- |
| 3 | 2 | 1 | 0 | -1 | -2 | -3 |

** Prefer not to answer*

1. **Is there anything else you would like to share about your preferences regarding this chronic disease group?**
   - ____________________________________________________________________________________________________________________________________________________________________________________________________________________________________________________________________________________________
   - Prefer not to answer

Before you are redirected to the exit survey to input your contact information for the random draw for 1 of 10 gift cards of $25, we would like to ask you for some demographic information. Collecting demographic information enables us to see if differences in group treatment preferences exist based on personal characteristics such as gender identity or racial background. It also helps us to understand if there are any gaps in our data to ensure that the information we have is as representative as possible.

1. **What is your age?**
   - _______________
   - Prefer not to answer
2. **What is your gender (for example, man, woman, non-binary, etc.)?**
   - _______________
   - Prefer not to answer
3. **We know that race is a social construct, and that race does not result in biological (including genetic) differences. However, our racial background still has important consequences for us, including how we are treated by individuals, groups, and institutions. What is your racial background (for example, Arab, Black, Indigenous, Chinese, Filipino, South Asian, White, etc.)?**
   - _______________
   - Prefer not to answer
4. **Where do you live in Saskatchewan?**
   - Regina
   - Saskatoon
   - Prince Albert
   - Moose Jaw
   - Other urban area including Estavan, Flin Flon, Humboldt, Lloydminster, Martensville, Meadow Lake, Melfort, Melville, North Battleford, Swift Current, Warman, Weyburn, and Yorkton
   - Rural area such as Arborfield, Buffalo Narrows, Indian Head, La Ronge, Radville, Shellbrook, Tisdale, Willow Creek, etc.
   - Prefer not to answer

Thank you for taking the time to participate in this survey!

Given the nature of the topic under study, some of the questions asked may have been distressing to you. As a reminder, if you experienced significant distress during or following your participation in this study, we encourage you to call your local crisis helpline or emergency department. Additional resources include Wellness Together Canada (<https://wellnesstogether.ca/en-CA>), the Canadian Mental Health Association (<https://cmha.ca/>), and the Mental Health Commission of Canada (<https://www.mentalhealthcommission.ca/>).

**Exit Survey**

You have been redirected to this exit survey to enter your contact information to be entered in a random draw to win 1 of 10 Amazon e-gift cards of $25. This contact information will be kept separate from any of the responses that you provided as part of the study. Once the random draw is complete and winners of the Amazon e-gift cards have received their gift card of $25, all contact information will be permanently deleted.

First Name: ____________________

Email Address: ____________________
